# Brewing potential of strains of the boreal wild yeast *Mrakia gelida*

**DOI:** 10.3389/fmicb.2023.1108961

**Published:** 2023-02-09

**Authors:** Riikka Linnakoski, Tuula Jyske, Ronja Eerikäinen, Pyry Veteli, Marta Cortina-Escribano, Frederico Magalhães, Eila Järvenpää, Lotta Heikkilä, Mathias Hutzler, Brian Gibson

**Affiliations:** ^1^Natural Resources Institute Finland (Luke), Helsinki, Finland; ^2^VTT Technical Research Centre of Finland Ltd., Espoo, Finland; ^3^Institute of Food Technology and Food Chemistry, Chair of Brewing and Beverage Technology, Technische Universität Berlin, Berlin, Germany; ^4^Natural Resources Institute Finland (Luke), Jokioinen, Finland; ^5^Research Centre Weihenstephan for Brewing and Food Quality, Technical University of Munich, Berlin, Germany

**Keywords:** Finnish forests, low-alcohol beer, tree, non-*Saccharomyces*, brewing yeast

## Abstract

Demand for low- or non-alcoholic beers has been growing in recent years. Thus, research is increasingly focusing on non-*Saccharomyces* species that typically are only able to consume the simple sugars in wort, and therefore have a limited production of alcohol. In this project, new species and strains of non-conventional yeasts were sampled and identified from Finnish forest environments. From this wild yeast collection, a number of *Mrakia gelida* strains were selected for small-scale fermentation tests and compared with a reference strain, the low-alcohol brewing yeast *Saccharomycodes ludwigii*. All the *M. gelida* strains were able to produce beer with an average of 0.7% alcohol, similar to the control strain. One *M. gelida* strain showing the most promising combination of good fermentation profile and production of desirable flavor active compounds was selected for pilot-scale (40 L) fermentation. The beers produced were matured, filtered, carbonated, and bottled. The bottled beers were then directed for in-house evaluation, and further analyzed for sensory profiles. The beers produced contained 0.6% Alcohol by volume (ABV). According to the sensory analysis, the beers were comparable to those produced by *S. ludwigii*, and contained detectable fruit notes (banana and plum). No distinct off-flavors were noted. A comprehensive analysis of *M. gelida*’s resistance to temperature extremes, disinfectant, common preservatives, and antifungal agents would suggest that the strains pose little risk to either process hygiene or occupational safety.

## 1. Introduction

Beer is the most consumed alcoholic beverage in the world. The global beer market reached US$ 554.65 billion in 2021 and it is expected to grow annually by compound annual growth rate (CAGR) 10.15% from 2022 to 2025 (Statista, 2022a). The global non-alcoholic beer market is expected to grow even faster, annually by CAGR 13.44% during the same time. In 2021, the non-alcoholic beer market generated revenue of roughly US$ 25.28 billion (Statista, 2022b).

According to Eurostat (2021) in 2020 almost 32 billion liters of beer containing alcohol and 1.4 billion liters of beer containing less than 0.5% alcohol or no alcohol content at all were produced in the EU. Non-alcoholic and low-alcoholic beers had a volume share of more than 10 percent in Spain and Germany in 2019, making them the countries where the beverages were the most popular (Statista, 2021b).

The beer market is driven by the increasing demand for premium and craft beers. Those are preferred because of their authenticity, taste, and aroma (Morgan et al., 2020). At the same time, increasing awareness of the negative health effects of alcohol consumption and rising adoption of healthy lifestyles, including weight management, are driving the growth of non- and low-alcohol beer market (Chrysochou, 2014; Gernat et al., 2020). Additionally, during the last few years technological developments have improved the flavor of non-alcoholic beer (Gernat et al., 2020; Melton, 2022), further increasing the market growth since many consumers are looking for products that taste similarly to their alcoholic equivalents (Muller et al., 2020).

The growing interest in low- and non-alcoholic beers has inspired research into novel or improved methods of production (Bellut and Arendt, 2019). These methods can broadly be described as physical or biological methods. Of these, the former approach, which includes membrane filtration and vacuum distillation, requires considerable investment in facilities. Biological methods, which include modified mashing procedures or limited fermentation processes are generally considered more cost effective and can also produce beers that more closely match those produced in conventional brewing. From the various methods that involve limiting fermentation, the use of non-conventional yeasts is gaining considerable interest (Methner et al., 2019; Statista, 2021a). These yeasts often utilize only simple sugars (glucose and fructose), but are unable to consume the dominant sugars found in brewer’s wort (maltose and maltotriose). With such yeasts there is therefore a natural limit to the extent of fermentation that occurs, and consequently the amount of alcohol that forms. Such yeasts have been isolated from multiple sources including other fermentation systems like kombucha or sourdough (Bellut et al., 2020), or even from the native brewery microbiota (Krogerus et al., 2021).

Many different yeast species have been considered for the production of low-alcohol beers, though they have invariably been members of the Ascomycota (Yabaci Karaoglan et al., 2022), presumably due to their ability to tolerate alcohol and anaerobic conditions. One exception is the yeast species *Mrakia gelida*, a member of the Basidiomycota. This species has shown potential for brewing (De Francesco et al., 2018), with beers rated well by a sensory panel compared to a reference brewing yeast, *Saccharomycodes ludwigii*, a yeast which has been used for over a century for the production of low-alcohol beers (Bellut and Arendt, 2019).

When introducing new strains to the brewing process, it is advisable to assess the potential risks to the process or to occupational safety. Relevant parameters in this regard include potential for production of yeast biofilm, resistance to disinfectants, preservatives and antibiotics, and ability to grow at different temperatures. All these properties affect the functionality and handling of yeasts in production. Strains that are better able to produce biofilm, grow at higher temperatures (e.g., at 37°C), and tolerate preservatives as well as disinfectants and antibiotics are potentially a greater risk to the brewing process and to human health.

A particularly interesting feature of *M. gelida* is that the species is psychrophilic. Low temperatures are typically employed in breweries when producing lager beers. The low temperature tolerance of *M. gelida* makes it compatible with current commercial brewing processes. In contrast, many non-conventional yeasts tested for low-alcohol beer production are mesophilic and require relatively warm temperatures for fermentation. Maintaining low temperatures during the whole brewing process also limits the likelihood of contamination–a considerable risk with low-alcohol beers, which contain relatively high levels of sugar as well as alcohol concentrations that do not inhibit contaminant growth. The fact that *M. gelida* does not grow at room temperature (i.e., above ca. 15°C) is also potentially an advantage in terms of brewery hygiene, i.e., simple steam treatments are sufficient for cleaning vessels, and there is little risk of cross-contamination within breweries.

The objectives of this study therefore were, firstly, to assess the brewing potential of a range of *M. gelida* strains of Finnish forest environment origin *via* small-scale fermentations and select a strain suitable for scaled-up fermentations; and secondly, perform a pilot-scale fermentation with the selected strain and a reference strain (*S. ludwigii*), after which bottled beers would be prepared for sensory evaluation.

## 2. Materials and methods

### 2.1. Collection and maintenance of yeast isolates

The *M. gelida* strains were collected during a larger survey conducted in Punkaharju experimental forests in Finland in late March 2019. In total, nine hardwood tree species and 10 individuals of each tree species were sampled. The yeasts were isolated using a modified method by Sniegowski et al. (2002), which has been used previously for the isolation of yeasts from tree bark. In the field, bark samples approximately 0.5 × 0.5 cm from around the bases of trees (at breast height, i.e., 1.3 m on the stem from tree stump) were cut using a sterile scalpel and tweezers and placed in 2.5 mL Eppendorf tubes containing sterile tap water.

The samples were stored in a freezer and transferred to laboratory and stored for up to 3 days at 4°C before enrichment culturing. In the laboratory, the bark samples were transferred to 50 mL Falcon tubes containing 5 mL of a sterile liquid enrichment medium [Yeast Malt (YM) Broth] consisting of 3 g yeast extract, 3 g malt extract, 5 g peptone and 10 g dextrose per liter. The YM Broth was acidified to pH 3.5 prior to autoclaving. The tubes were then incubated for up to 3 weeks at 5°C with shaking (80 rpm) and inspected for signs of proliferation of yeasts or other cells. Aliquots of 10 μL from each of these liquid enrichment cultures were then streaked on solid YM agar medium containing the same ingredients as the YM Broth and 20 g agar per liter. Plates were incubated for several days at 5°C and examined regularly for any growth of yeast or other colonies. Purified cultures were obtained by picking colonies and re-streaking them onto to fresh YM Agar medium. The cultures are stored at the culture collection of Natural Resources Institute Finland (Luke), Helsinki.

### 2.2. Identification of yeasts

The isolated strains were identified based on the internal transcribed spacer (ITS) gene region sequences. The DNA was extracted using PrepMan™ Ultra Sample preparation reagent (Applied Biosystems, Foster City, CA, USA) following the manufacturer’s protocol. The ITS gene region was amplified, and sequencing performed using a primer pair ITS1-F (Gardes and Bruns, 1993) and ITS4 (White et al., 1990). The PCR reaction mixture (25 μL volume) contained 0.2 μL of Phusion High-Fidelity DNA Polymerase (2 U μL^–1^) (Thermo Fisher Scientific, Waltham, MA, USA), 4 μL of Reaction Buffer (5 × Phusion HF), 0.4 μL of dNTPs (10 mM), and 0.5 μL of each primer. PCR reactions were performed as follows: an initial denaturation step at 98°C for 30 s, followed by 35 cycles of 5 s at 98°C, 10 s at 55°C and 30 s min at 72°C, and a final chain elongation at 72°C for 8 min. PCR products were visualized under UV light after staining 5 μL aliquots with ethidium bromide and separation on a 1% agarose gel. Successfully amplified products were purified using the Exo-SAP protocol: 20 μL of the PCR product was mixed with 8 μL of Exo-SAP [5 μL of Exonuclease I (20 U μL^–1^) (Fermentas, Vilnius, Lithuania) and 100 μL of Shrimp Alkaline Phosphatase (1 U μL^–1^) (Roche Diagnostics, Indianapolis, USA) in a 1,000 μL reaction mixture] and incubated at 37°C for 15 min and following immediate incubation at 80°C for 15 min. The sequencing was conducted at Macrogen Europe.

The forward and reverse sequences were assembled using Geneious 10.2.6 (Biomatters Ltd., Auckland, New Zealand). The obtained ITS sequences were initially identified using BLASTn search^1^ (National Center for Biotechnology Information, U.S. National Library of Medicine, Bethesda MD, USA) in the NCBI nucleotide (nt) database. The identification of the obtained *Mrakia* isolates was further confirmed by three phylogenetic methods: maximum likelihood (ML), maximum parsimony (MP), and Bayesian inference (BI) as described earlier by Linnakoski et al. (2021). The dataset including type sequences and sequences of closed related sequences was compiled with MEGA v.7 (Kumar et al., 2016) and aligned using the online version of MAFFT v.7 (Katoh and Standley, 2013) with the FFT-NS-i strategy. The sequence data generated in this study has been deposited in GenBank (Table 1).

**TABLE 1:** *Mrakia gelida* strains included in the present study.

Luke culture number	Species	Host tree	GenBank Acc. no
YGW70	*Mrakia gelida*	*Quercus robur*	OM604737
YGW132	*Mrakia gelida*	*Sorbus aucuparia*	OM604738
YGW150	*Mrakia gelida*	*Tilia cordata*	OM604739
YGW172	*Mrakia gelida*	*Ulmus laevis*	OM604740
YGW180	*Mrakia gelida*	*U. laevis*	OM604741
YGW184	*Mrakia gelida*	*U. laevis*	OM604742
YGW279	*Mrakia gelida*	*Q. robur*	OM604743
YGW321	*Mrakia gelida*	*Q. robur*	OM604744
YGW322	*Mrakia gelida*	*Q. robur*	OM604745
YGW335	*Mrakia gelida*	*Populus tremula*	OM604746
YGW344	*Mrakia gelida*	*P. tremula*	OM604747
YGW352	*Mrakia gelida*	*P. tremula*	OM604748

### 2.3. Selection of yeast strains for brewing trials

Table 1 shows the *M. gelida* strains selected for the present study.

### 2.4. Brewing process

#### 2.4.1. Mashing process

Wort was prepared using malt from Viking Malt Oy (Lahti, Finland) (90% pilsner malt and 10% Vienna malt). 26 kg of malt (hammer milled) was used to produce approx. 1 hL of 15°P wort. 3 L of water were added per kg malt. The mash was supplemented with 52 mL of 85% lactic acid [this is a standard adjustment for the brews prepared at VTT. The pH was not adjusted to a level typical for finished beers (< pH 4.5)], 30 g CaCl.2H_2_O, 10 g CaSO_4_.2H_2_O, and 53 mg ZnSO_4_.7H_2_O. Mash profile was as follows: 48°C, 30 min; 63°C, 30 min; 72°C, 30 min and 78°C, 10 min. A Meura filter was used for wort separation. Wort was boiled for 60 min with 113 g Magnum hops (15% alpha acid, target IBU 45). Hot trub was removed by whirlpool. The wort was then diluted with deaerated water 50:50 to achieve 7.5°P prior to the fermentation.

#### 2.4.2. Fermentation process

Strains of *S. ludwigii* (C-79089, cider isolate, NCYC collection) and *M. gelida* were propagated in 50 mL YP medium containing 2% (w/v) glucose. *S. ludwigii* was grown for 24 h at room temperature (at about 20°C; also, from herein when referred to room temperature), while *M. gelida* strains were grown for 48 h at 15°C. 20% (w/v) yeast slurries were prepared with distilled water for each strain and cell numbers evaluated with the aid of the Chemometec Nucleocounter. A total of 100 mL of 7.5°P brewer’s wort in a 250 mL Erlenmeyer flask was inoculated at a rate of 10 million cells mL^–1^. Airlocks were attached to maintain anaerobic conditions. Fermentations proceeded at 10°C for 14 days and fermentation progress was monitored *via*%-mass loss from the wort. The *S. ludwigii* fermentations were carried out at 10°C or 20°C. For 40 L –scale fermentations, *S. ludwigii* C-79089 and *M. gelida* YGW184 were propagated in 1 L Yeast extract, Peptone, dextrose (YPD) and subsequently 10 L YPD at 15°C. Yeast slurries were prepared as before and inoculated into 40 L of 7.5°P wort. Fermentations proceeded at 10°C for 6 days, before maturation for 8 days at 4°C.

After maturation, beers were depth-filtered (Seitz EK, Pall Corporation, Port Washington, NY, USA) and were assessed for alcohol content, wort density, pH, and yeast content (mass in suspension). Filtered beers were carbonated to approx. 5 g L^–1^ CO_2_. Beers were then transferred to brown, 330 mL bottles and stored cold (about +10°C to +12°C) until sensory evaluation.

### 2.5. Analytics

The collected samples of wort or beer were centrifuged, and supernatants used for analyses after manual degassing. The specific gravity, alcohol level (% v/v) and pH of samples were determined from the centrifuged and degassed wort and fermentation samples using an Anton Paar Density Meter DMA 5000 M with Alcolyzer Beer ME and pH ME modules (Anton Paar GmbH, Austria).

Yeast-derived aroma compounds (acetaldehyde, higher alcohols, and esters) were determined by headspace gas chromatography with flame ionization detector (HS-GC-FID) analysis. 4 mL samples were filtered (0.45 μm), incubated at 60°C for 30 min and then 1 mL of gas phase was injected (split mode; 225°C; split flow of 30 mL min^–1^) into a gas chromatograph equipped with an FID detector and headspace autosampler (Agilent 7890 Series; Palo Alto, CA, USA). Analytes were separated on a HP-5 capillary column (50 m × 320 μm × 1.05 μm column, Agilent, USA). The carrier gas was helium (constant flow of 1.4 mL min^–1^). The temperature program was 50°C for 3 min, 10°C min^–1^ to 100°C, 5°C min^–1^ to 140°C, 15°C min^–1^ to 260°C and then isothermal for 1 min. Compounds were identified by comparison with authentic standards and were quantified using standard curves. 1-butanol was used as internal standard.

Concentration of fermentable sugars (glucose, fructose, maltose, and maltotriose) in bottled beers was measured by high-performance liquid chromatography (HPLC) using a Waters 2,695 separation module and Waters system interface module liquid chromatograph coupled with a Waters 2,414 differential refractometer (Waters Co., Milford, MA, USA). An Aminex HPX-87H organic acid analysis column (300 × 7.8 mm, Bio-Rad Inc., Hercules, CA, USA) was equilibrated with 5 mM H_2_SO_4_ (Titrisol, Merck, Germany) in water at 55°C, and samples were eluted with 5 mM H_2_SO_4_ in water at a 0.3 mL min^–1^ flow rate.

Aldehydes were analyzed as oximes by using a headspace sampler (Agilent 7697A) coupled with gas chromatograph (Agilent 7890B) and compounds were detected using a Micro Electron Capture Detector (HS-GC-ECD). Carbonyl compound standards were 2-methylpropanal, 2-methylbutanal, 3-methylbutanal, hexanal, furfural, methional, phenylacetaldehyde, and (E)-2-non-enal (Sigma-Aldrich, St. Louis, MO, USA). A stock solution containing a mixture of the standard compounds in ethanol was prepared at 1,000 μg L^–1^ each. The calibration range was 0.5–40 μg L^–1^ and dilutions were prepared in 5% ethanol. The sum of the peak areas of the two geometrical isomers (E and Z) was used for calculations. Correlation coefficient (R^2^) values were 0.995–0.9999. An aqueous solution of derivatization agent O-(2,3,4,5,6-pentafluorobenzyl)-hydroxylamine (PFBOA) (Sigma-Aldrich) was prepared at a concentration of 6 g L^–1^. One hundred microliters of this solution and 5 mL of deionized water or beer were placed in a 20-mL glass vial and sealed with a crimp cap (Agilent Technologies Inc., Santa Clara, CA, USA). The sample/standard vial was then placed in the headspace sampler with following conditions: sample equilibrium in oven for 30 min at 60°C, after which 1 min of injection of sample fill pressurized at 25 psi. Loop temperature was 100°C and transfer line was held at 110°C. The following GC conditions were applied: HP-5 capillary column, 50 m × 0.32 mm × 1.05 μm (J&W Scientific, Folsom, CA, USA). Helium was the carrier gas at a flow rate of 1.0 mL min^–1^ and for ECD, nitrogen make up gas was applied at a flow rate of 30 mL min^–1^. The front inlet temperature was 250°C. The injection was in the split mode and the split ratio 10:1 was applied. The oven temperature program used was 40°C for 2 min, followed by an increase of 10°C min^–1^ to 140°C (held 5 min) and 7°C min^–1^ to 250°C. The final temperature was held for 3 min.

### 2.6. Sensory analysis

Bottled beer samples (matured as described in Section “2.4.2. Fermentation process”) were tasted and judged by a trained sensory panel of seven panelists certified by the Deutsche Landwirtschafts-Gesellschaft (DLG, Frankfurt, Germany). Tasting was performed in a dedicated tasting room (individual tasting chambers, white-colored room, no distracting influences, and brown glasses with three-digit number labels) to exclude all external misleading factors. The main flavor impressions were determined at a range from 1 (almost no perception) to 5 (very high perception). Flavor impressions were chosen according to Meier-Dörnberg et al. (2017). In addition, a tasting was performed under the same circumstances with the DLG scheme, in which the beer is judged by its aroma, taste, carbonation, body, and bitterness in a range of 1–5, 1 being the lowest value (negative) and 5 being the highest value (positive).

### 2.7. Hygiene and safety of strains

These experiments tested the tolerance of *M. gelida* (YGW 184 strain) to common preservatives [isomerized hop extract IsoHop^®^ 30 IBU (BarthHaas, Nuremberg, Germany), ethanol 5% (v/v) (AaS, Rajamäki, Finland), benzoate 150 mg L^–1^ (sodium benzoate, Sigma-Aldrich, Darmstadt, Germany), sorbate 250 mg L^–1^ (potassium sorbate, Sigma-Aldrich, Darmstadt, Germany), and sulfite 200 mg L^–1^ (potassium metabisulphite, Brown, Poland)], antibiotics (Anidulafungin, Amphotericin B, Micafungin, Caspofungin, 5-Flucytosine, Posaconazole, Voriconazole, and Itraconazole and Fluconazole) and disinfectant [P3-oxonia active (Oy Ecolab Ab, Helsinki, Finland)], its ability to grow at different temperatures, and its ability to form a biofilm in static and agitated conditions. Known brewer’s yeasts *Saccharomyces pastorianus* (lager) and *S. ludwigii* (non-alcoholic) strains were used as references. *M. gelida* is a well-known psychrophile, so tests were carried out at suitable temperatures (i.e., +1°C, +4°C, and +37°C). At the same time, the behavior of the reference yeasts under these conditions was tested.

#### 2.7.1. Biofilm-forming potential

Yeast biofilm production was tested using 96-well microplates. Strains were propagated by taking a loopful of fresh yeast mass from YPD agar and inoculating into 25 mL of liquid YPD and incubating for 1 day on a shaker (120 rpm) at room temperature. Grown cell cultures’ optical densities (OD600) were measured with a spectrophotometer (UV-1800, Shimadzu Corporation, Kyoto, Japan). Based on the lowest optical density, the yeasts were diluted for growth. Most wells were filled with 250 μL of 10°P inoculation wort, including wells for yeasts and for blank samples. Remaining wells were empty or filled with water. 2.5 μL of culture was used to inoculate 10 replicate wells. Growing times were one or 4 days, with either slight shaking or without agitation. Assays were performed at 13°C. After 24 h or 4 days, OD600 values were measured from each yeast from one well, to be sure there has been growth. Biofilm-forming potential was assessed by measuring attachment of the cells to the walls of the wells. The plate was first rinsed with sterile Milli-Q-filtered water. After which, 300 μL of 0.1% crystal violet solution was placed in the wells for 5 min. It was then rinsed in the collection vessel three times with sterile Milli-Q-filtered water. The plate was left to air-dry for 15 min in a laminar flow cabinet. The remaining crystal violet, which was still bound to the cells, was dissolved with 300 μL of 95% Etax B (AaS, Rajamäki, Finland). Absorbances of the wells were measured at 595 nm with a Multiskan EX (Labsystems Oy, Finland).

#### 2.7.2. Temperature tolerance

The ability of the yeasts to grow at different temperatures was tested using a spot plate technique. Yeasts were propagated by taking a loopful of fresh yeast mass from YPD agar and inoculating into 25 mL of liquid YPD for 1 day on a shaker (120 rpm) at room temperature. Each culture was centrifuged, washed, and re-suspended to OD600 of 0.1 (2 × 106 cells mL^–1^) and further diluted to concentrations of 0.01, 0.001, and 0.0001 using sterile Milli-Q-filtered water. 5 μL of each suspension was spotted onto the surface of a YPD agar plate. The plates were incubated at 37°C for 3 days, 4°C for 2 weeks, and 1°C for 3 weeks.

#### 2.7.3. Preservative tolerance

The tolerance of the yeasts to common food preservatives was assessed in microplate cultivations using a Bioscreen C incubator and plate reader (Labsystems Oy, Finland). Yeasts were propagated by taking a loopful of fresh yeast mass from YPD agar and inoculating into 25 mL of liquid YPD for 1 day on a shaker (120 rpm) at room temperature. The culture was then centrifuged at 9,000 rpm for 5 min at 4°C. The pellet was washed with 10 mL of sterile physiological saline solution (0.9% NaCl). A 20%-slurry was prepared, and cell density was measured with a NucleoCounter YC-100 (Chemometec, Denmark). YPD media were adjusted to pH 4 with hydrogen chloride (HCl) before use. The microplate’s wells were filled with 150 μL YPD (1% glucose w/v, pH 4) and with 140 μL of one of the following preservatives to complete these final volumes: isomerized hop extract IsoHop^®^ 30 IBU (BarthHaas, Nuremberg, Germany), ethanol 5% (v/v) (AaS, Rajamäki, Finland), benzoate 150 mg L^–1^ (sodium benzoate, Sigma-Aldrich, Darmstadt, Germany), sorbate 250 mg L^–1^ (potassium sorbate, Sigma-Aldrich, Darmstadt, Germany), and sulfite 200 mg L^–1^ (potassium metabisulfite, Brown, Poland). A total of 20% yeast slurry was diluted to obtain approximately 2,000 cells per well (by using NucleoCounter YC-100 results and adding dilution at 10 μL per well). The final volume was 300 μL per well, and the cultivations were carried out at 13°C with moderate shaking. Each culture was prepared in triplicate for each preservative.

#### 2.7.4. Disinfectant tolerance

The tolerance of yeasts to the disinfectant P3-oxonia active (Oy Ecolab Ab, Helsinki, Finland) was tested as follows. YPD agar plates were prepared with suspensions with yeast and bovine serum albumin (BSA) (Sigma-Aldrich, Missouri, United States). One concentration of BSA represented “clean” conditions (0.3% BSA), and one represented “unclean” condition (3% BSA). BSA dilutions were made with sterile milli-Q-filtered water. Yeast suspensions were prepared by taking a loopful of fresh yeast mass from a YPD agar plate culture and inoculating into 5 mL of sterile 0.9% NaCl solution. The yeast suspension was added at 60 μL for both conditions, to 3 mL of 0.3% BSA and to 3 mL of 3% BSA. Each yeast was added to test agar plates in duplicate for each sample time. Before adding disinfectant, both suspensions with yeast were spread on test plates for controls. The disinfectant was added at 300 μL (at a final concentration of 0.3%) for both conditions, and timing started after addition. Times were 2.5, 5, 10, and 20 min. At each time point, solutions were mixed well by vortexing [to obtain a condition similar to the cleaning in place (CIP) method] and transferred to an agar plate. The plates were incubated at 25°C, except for *M. gelida* at 10°C, for three to 5 days, depending on the growth yield. Resistance to the P3-oxonia was assessed qualitatively depending on the relative growth of the yeasts.

#### 2.7.5. Antibiotic resistance

*Mrakia gelida* is a psychrophile and does not therefore pose a direct risk to human health. To further ensure handling safety, the species’ resistance to antibiotics was assessed. Resistance was evaluated using the YeastOne YO10 (Thermo Fischer) test plate according to the manufacturer’s instructions. The antifungal test included nine commonly used antifungal agents: Anidulafungin, Amphotericin B, Micafungin, Caspofungin, 5-Flucytosine, Posaconazole, Voriconazole, and Itraconazole and Fluconazole. The test was performed at 13°C for 3 days.

### 2.8. Statistical analysis

Linear mixed models were used to compare the mean concentrations of key flavor active volatiles present in the beers produced by using the *M. gelida* yeast strain YGW184 against those produced by the other *M. gelida* strains and *S. ludwigii* by using Dunnett’s pairwise comparison test. The assumption of unequal variances of treatments was allowed when necessary (based on a likelihood ratio test) and technical replicates were taken into account through a random effect. The assumption of normality of the residuals was studied graphically and was found to be adequate for all models.

## 3. Results

### 3.1. Isolation and identification of the yeasts

A total of 12 *M. gelida* strains isolated from the bark of five different host trees collected in Punkaharju, Finland, were identified by sequence similarity of the ITS gene region (Table 1). The accurate identity was further confirmed by phylogenetic analysis. Based on the phylogenetic analysis of the ITS region, the *M. gelida* strains collected in this study formed a clade with high bootstrap support value with the European isolate of *M. gelida* (GenBank acc. MK496827) and the type strain from Antarctica (GenBank acc. NR_163504) (Supplementary Figure 1).

### 3.2. Small-scale fermentation performance

**FIGURE 1:**
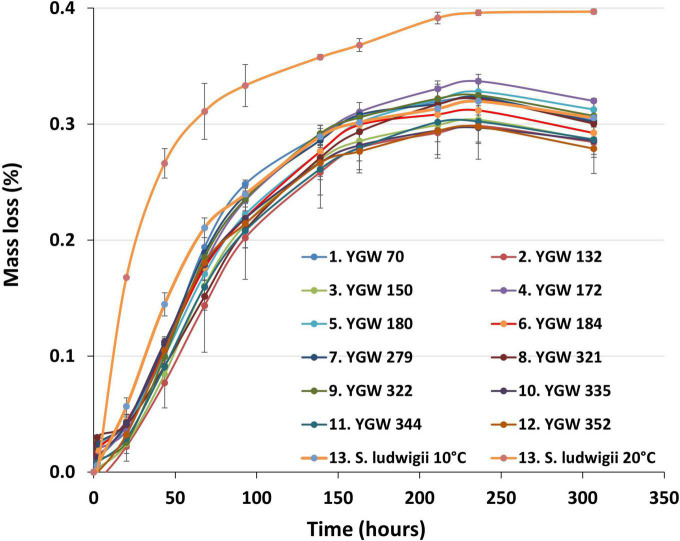
Small-scale fermentation progress as measured by mass loss from 7.5°P wort fermented with strains of *Mrakia gelida* at 10°C. Reference conditions include *Saccharomycodes ludwigii* incubated at 10°C and 20°C. Values are means of two independent replicate fermentations (± standard deviation).

Fermentation progress, as measured by mass loss, was similar for all *M. gelida* strains (Figure 1). There was no indication of any substantial lag phase before fermentation started, as all strains showed some evidence of fermentation after 20 h. Performance of the reference *S. ludwigii* strain at 10°C was marginally better than the test strains in the first 72 h of fermentation. This advantage was not, however, apparent after this time. When the reference strain was incubated at 20°C, fermentation was considerably faster and more efficient. Test strains did not meet the same level of fermentation despite their reported psychrophilic nature. For test strains the% mass loss varied from 0.28 to 0.32%. Values were considerably lower than would be expected with a production yeast strain, where values of approx. 5% mass loss would be typical. Results were consistent with only monosaccharide sugars being utilized.

Alcohol levels were also correspondingly low, with values ranging from 0.69 to 0.74% Alcohol by volume (ABV). Highest alcohol level was seen with the reference strain fermentations at 20°C (0.78%), consistent with the higher mass loss values seen with this yeast (Table 2 and Figure 1). pH of the beer was approx. 4.8 for all strains (Table 2). This relatively high value is typical for low-alcohol beers and can readily be corrected to a normal beer pH value (approx. 4.4) through addition of lactic acid to wort or beer.

**TABLE 2:** Alcohol content and pH of *Mrakia gelida* beers, and yeast fresh mass and yeast viability after fermentation of 7.5°P wort for 2 weeks at 10°C.

Yeast	Alcohol (% v/v)	pH	Yeast fresh mass (g L^–1^)	Yeast viability (%)
YGW 70	0.75 (0.01)	4.81 (0.02)	14.5 (0.26)	94.9 (0.2)
YGW 132	0.71 (0.01)	4.76 (0.00)	14.2 (0.23)	93.0 (1.0)
YGW 150	0.70 (0.00)	4.78 (0.00)	15.6 (0.15)	93.0 (0.0)
YGW 172	0.75 (0.01)	4.78 (0.01)	14.5 (0.10)	93.9 (0.0)
YGW 180	0.73 (0.01)	4.76 (0.00)	15.2 (0.60)	95.2 (1.0)
**YGW 184**	0.70 (0.00)	4.75 (0.00)	15.6 (0.56)	92.7 (0.0)
YGW 279	0.72 (0.01)	4.96 (0.10)	16.1 (0.11)	91.1 (0.0)
YGW 321	0.71 (0.00)	4.75 (0.00)	14.0 (0.33)	92.4 (0.0)
YGW 322	0.75 (0.02)	4.77 (0.00)	16.1 (0.57)	94.7 (0.9)
YGW 335	0.69 (0.01)	4.77 (0.00)	15.8 (0.97)	92.9 (0.0)
YGW 344	0.71 (0.00)	4.75 (0.00)	13.5 (0.46)	97.2 (0.0)
YGW 352	0.73 (0.01)	4.75 (0.02)	15.9 (1.25)	89.0 (0.2)
*S. ludwigii* 10°C	0.76 (0.02)	4.75 (0.00)	25.8 (0.87)	91.1 (0.0)
*S. ludwigii* 20°C	0.78 (0.00)	4.82 (0.01)	29.8 (0.99)	94.9

Reference conditions include *Saccharomycodes ludwigii* incubated at 10°C and 20°C. Values are means of two independent replicate fermentations (range in parenthesis). Viability value for *S. ludwigii* at 20°C could not be calculated due to formation of recalcitrant flocs. The strain in bold refers to the one that was selected for the larger scale fermentation.

Yeast fresh mass was consistent amongst the test strains, with values all falling in the range of 13.5–16.1 g L^–1^ (Table 2). By contrast, the reference yeast produced 26 g L^–1^ and 30 g L^–1^ after incubation at 10°C and 20°C, respectively.

All strains maintained a high viability (>90%) indicating, firstly, that the fermentation conditions were not particularly stressful, and secondly, that the yeast at the end of fermentation would be suitable for repitching, i.e., re-use in subsequent fermentations–a standard brewery practice (Table 2). The *S. ludwigii* yeast from the 20°C fermentation was in a flocculent state which prevented the viability assay from being carried out in this case.

Analysis of flavor volatiles (acetaldehyde, higher alcohols, and esters) showed a relatively low level of yeast-derived flavor compounds in all beers (Table 3). No compounds were present above normally recognized flavor threshold values (Meilgaard, 1975). Acetaldehyde levels in test beers were close to the flavor threshold of 25 mg L^–1^, though the grassy flavor imparted by acetaldehyde is not generally seen as a positive flavor attribute. Acetaldehyde values were considerably lower in the reference beers (<1.0 mg L^–1^). Based on its slightly higher production of flavor volatiles, the *M. gelida* strain YGW 184 was chosen for scaled-up fermentations. The statistically significant differences in concentrations of flavor volatiles between the YGW 184 as compared to other strains are shown in Table 3.

**TABLE 3:** Mean concentrations (± range) of key flavor active volatiles present in the beers produced by using the *Mrakia gelida* yeast strains and *Saccharomycodes ludwigii*.

	Concentrations (mg L^–1^)					
**Yeast**	**Acetaldehyde**	**Propanol**	**Ethyl acetate**	**2-Methyl-propanol**	**3-Methyl-butanol**	**2-Methyl-butanol**
YGW 70	20.76 (1.19)	^b^7.68 (0.05)	^b^0.04 (0.00)	^b^9.67 (0.28)	^b^5.35 (0.23)	1.31 (0.05)
YGW 132	19.26 (1.41)	^b^6.95 (0.09)	^b^0.03 (0.00)	6.10 (0.07)	^b^6.47 (0.17)	1.40 (0.01)
YGW 150	19.42 (0.33)	^b^5.92 (0.01)	^b^0.06 (0.02)	6.14 (0.03)	7.57 (0.01)	^b^1.59 (0.02)
YGW 172	^b^17.58 (0.46)	^b^7.87 (0.01)	0.14 (0.01)	6.36 (0.09)	^b^6.19 (0.09)	1.41 (0.01)
YGW 180	22.39 (1.13)	^b^6.34 (0.01)	0.14 (0.00)	5.92 (0.14)	^b^5.79 (0.13)	1.27 (0.03)
**YGW 184**	^a^22.24 (0.69)	^a^8.82 (0.10)	^a^0.20 (0.05)	^a^6.38 (0.28)	^a^8.34 (0.28)	^a^1.33 (0.02)
YGW 279	22.53 (0.04)	8.26 (0.15)	0.14 (0.00)	6.84 (0.21)	9.34 (0.33)	1.49 (0.04)
YGW 321	^b^16.55 (0.28)	^b^11.1 (0.12)	0.17 (0.03)	^b^8.09 (0.13)	8.96 (0.05)	^b^1.48 (0.02)
YGW 322	20.14 (0.86)	9.12 (0.04)	0.11 (0.06)	^b^11.11 (0.05)	^b^5.99 (0.09)	^b^1.08 (0.02)
YGW 335	17.66 (2.57)	8.32 (0.02)	^b^0.04 (0.01)	6.01 (0.12)	8.41 (0.33)	^b^1.16 (0.02)
YGW 344	19.19 (1.96)	^b^6.51 (0.11)	^b^0.04 (0.00)	5.83 (0.02)	^b^6.03 (0.02)	^b^1.21 (0.00)
YGW 352	^b^13.91 (1.84)	7.57 (0.59)	^b^0.04 (0.00)	6.81 (0.18)	8.57 (0.63)	1.35 (0.02)
*S. ludwigii* 10°C	^b^0.56 (0.01)	^b^2.25 (0.01)	^b^0.54 (0.00)	^b^10.93 (0.01)	^b^27.63 (0.05)	^b^3.68 (0.01)
*S. ludwigii* 20°C	^b^0.76 (0.07)	^b^2.36 (0.03)	^b^0.59 (0.01)	^b^12.51 (0.13)	^b^20.77 (0.22)	^b^4.63 (0.04)

The values are means of two independent replicates. Statistically significant differences (*p* < 0.05) in concentrations between those of YGW 184 as compared to those of other strains are indicated with the lower-case letter b. Lower-case letter a refers to the strain that was used in pair-wise comparisons. The strain in bold is the one selected for the larger scale fermentation.

Many wort aldehydes were present at high levels prior to fermentation, but most were reduced to levels lower than flavor thresholds in the beers. This was particularly the case for the *M. gelida* strains, which were more efficient reducers of aldehydes compared to *S. ludwigii*. An important aldehyde, methional (cooked potato aroma) still above the flavor threshold after fermentation with *S. ludwigii* but was below the threshold in all the *M. gelida* beers (Table 4).

**TABLE 4:** Concentrations of aldehydes (μg L^–1^) present in beers, and in the original wort.

	Concentrations (μg L^–1^)						
**Yeast**	**2-Methyl- propanal**	**2-Methyl –butanal**	**3-Methyl-butanal**	**Furfural**	**Methional**	**Phenyl acetaldehyde**	**Benz-aldehyde**
YGW 70	3.7 (0.1)	0.2 (0.0)	0.3 (0.0)	0.2 (0.0)	1.1 (0.0)	0.3 (0.0)	nd
YGW 132	5.3 (0.4)	0.3 (0.0)	0.6 (0.0)	0.3 (0.0)	1.5 (0.3)	0.6 (0.2)	nd
YGW 150	6.3 (0.5)	0.4 (0.0)	1.3 (0.2)	0.3 (0.1)	2.0 (0.5)	0.7 (0.1)	nd
YGW 172	5.1 (0.3)	0.3 (0.0)	0.6 (0.1)	0.3 (0.0)	1.5 (0.1)	0.5 (0.1)	nd
YGW 180	5.8 (0.5)	0.3 (0.0)	1.1 (0.0)	0.3 (0.0)	1.2 (0.3)	0.3 (0.1)	nd
**YGW 184**	4.5 (1.0)	0.3 (0.1)	0.7 (0.4)	0.3 (0.1)	1.0 (0.2)	0.4 (0.1)	nd
YGW 279	4.6 (0.7)	0.3 (0.1)	0.8 (0.5)	0.3 (0.0)	1.1 (0.2)	0.4 (0.0)	nd
YGW 321	3.7 (0.3)	0.2 (0.1)	0.3 (0.2)	0.3 (0.0)	1.5 (0.5)	0.6 (0.3)	nd
YGW 322	3.5 (0.9)	0.2 (0.0)	0.4 (0.2)	0.3 (0.1)	1.6 (0.4)	0.8 (0.4)	nd
YGW 335	3.9 (1.5)	0.2 (0.1)	0.5 (0.4)	0.3 (0.0)	1.5 (0.5)	0.6 (0.3)	nd
YGW 344	5.8 (0.0)	0.4 (0.0)	0.9 (0.1)	0.3 (0.0)	1.3 (0.1)	0.6 (0.2)	nd
YGW 352	7.3 (1.4)	0.5 (0.1)	2.4 (0.6)	0.3 (0.0)	3.1 (0.2)	1.5 (0.3)	nd
*S. ludwigii* 10°C	12.7 (0.8)	1.7 (0.1)	4.7 (0.0)	2.2 (0.0)	6.2 (0.3)	6.9 (0.1)	0.6 (0.0)
*S. ludwigii* 20°C	15.6 (2.3)	2.3 (0.0)	5.8 (0.1)	2.3 (0.1)	5.0 (0.2)	7.8 (0.5)	0.7 (0.1)
Wort	72.7 (15.4)	35.3 (1.6)	117.1 (23.2)	106.6 (8.0)	208.1 (6.2)	48.2 (4.0)	3.0

Values are means of two independent replicates. Values in bold are above flavor thresholds (Meilgaard, 1975; Saison et al., 2009). Deviation from the mean is indicated by range in parenthesis. nd, not detected.

### 3.3. Pilot-scale fermentation performance

**FIGURE 2:**
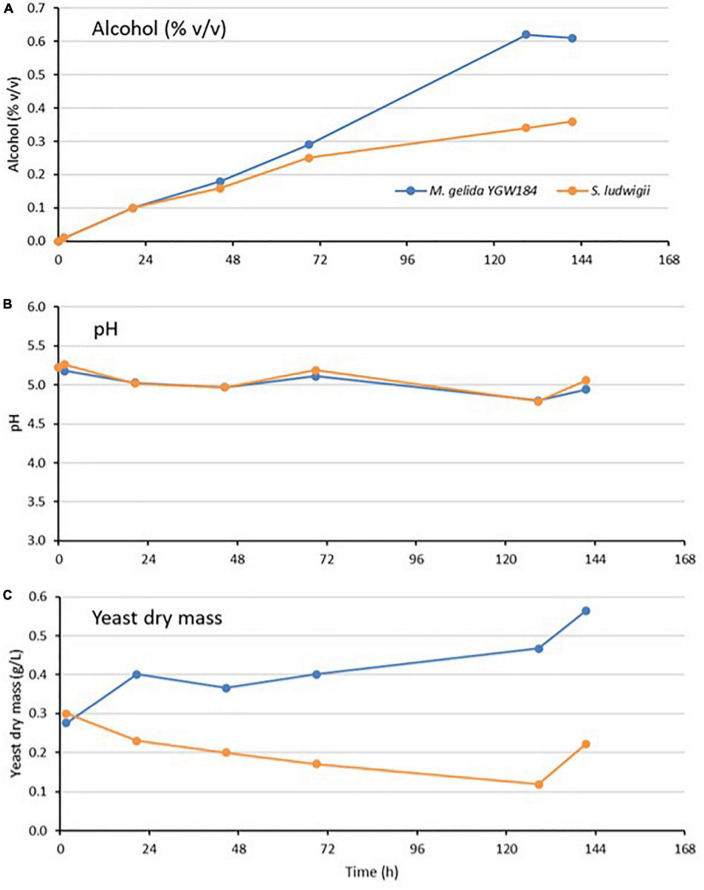
Fermentation of 7.5°P wort at 10°C by *Mrakia gelida* YGW184 and *Saccharomycodes ludwigii* measured by alcohol formation **(A)**, change in wort pH during fermentation **(B)**, and change in yeast dry mass in suspension during the fermentation **(C)**.

Pilot-scale (40 L) fermentations with *M. gelida* YGW 184 and the control strain *S. ludwigii* were carried out for 6 days at 10°C. Fermentation progress was assessed by daily measurement of the specific gravity, alcohol level (% v/v), pH, and yeast mass (Figure 2). Fermentation performance was identical in the first 24 h but from then on, the selected strain of *M. gelida* outperformed *S. ludwigii*, reaching an alcohol concentration of 0.61 versus 0.36% ABV at the end of the fermentation. Relative to the small-scale fermentation, the performance difference between the two strains was greater. *S. ludwigii* appeared to show relatively heavy sedimentation, as demonstrated by the low yeast mass in suspension during the fermentation (Figure 2).

### 3.4. Bottled beer analysis

The concentration of fermentable sugars remaining in the beers was quantified by HPLC. Neither strain was expected to consume any of the maltose and maltotriose present in the wort, however, there were differences in the final concentration between the strains (Table 5). This can be at least partially explained by the lower cell count obtained for *S. ludwigii* during propagation step (3.34 × 10^8^ cells mL^–1^ for *S. ludwigii* and 13.2 × 10^8^ cells mL^–1^ for *M. gelida*). This difference reflects in the volume of cell suspension needed for inoculation (1,198 mL for *S. ludwigii* and 304 mL for *M. gelida*). A higher inoculation volume diluted the sugar concentration in the wort. Besides this, beers produced with *M. gelida* beers had no residual glucose present (compared to 1.8 g L^–1^ in *S. ludwigii* beers) and half the concentration of fructose relative to *S. ludwigii*, consistent with *M. gelida*’s superior fermentation performance.

**TABLE 5:** Alcohol, pH, and sugar concentration in the bottled beers fermented with *Saccharomycodes ludwigii* and *Mrakia gelida* for 6 days at 10°C.

*S. ludwigii*		*M. gelida* YGW184
Alcohol (% v/v)	0.36	0.61
pH	5.1	4.9
**Sugars (g/L)**		
Maltose	31.1 (1.3)	35.1 (1.3)
Maltotriose	11.3 (0.4)	13.0 (0.6)
Glucose	1.8 (0.0)	ND
Fructose	1.8 (0.0)	0.8 (0.1)

The beers were matured for 8 days at 4°C before the analysis. Sugar values are means of two technical replicates (± standard deviation). ND, not detected.

The concentrations of flavor active aroma compounds in the beers were generally lower that those obtained in the small-scale fermentation (Table 6), with the exceptions being acetaldehyde and phenylethyl acetate in *S. ludwigii*. However, the concentrations were still lower than the flavor threshold (Meilgaard, 1975). The lower concentration of flavor compounds was likely explained by the shorter fermentation time and low temperature.

**TABLE 6:** Concentrations of the flavor active volatiles present in the bottled beers.

*S. ludwigii*		*M. gelida* YGW 184
Acetaldehyde	2.72 (0.34)	1.17 (0.19)
Propanol	0.88 (0.04)	4.58 (0.12)
Ethyl acetate	0.29 (0.03)	
2-Me-Propanol	5.10 (0.10)	3.38 (0.05)
3-Me-Butanol	13.50 (0.19)	5.16 (0.02)
2-Me-Butanol	2.06 (0.09)	0.70 (0.00)
3-Me-Butylacetate	0.01 (0.00)	ND
Ethyl hexanoate	ND	ND
Phenyl ethanol	7.22 (0.42)	ND
Ethyl octanoate	ND	ND
Phenylethylacetate	0.03 (0.05)	ND
Ethyl decanoate	ND	ND

Values are means of two technical replicates (± standard deviation). ND, not detected.

### 3.5. Sensory analysis

Beer “*S. ludwigii* bottled” was evaluated with grades above four according to the DLG tasting scheme (Table 7). Values below 4 indicate off-flavors. Hence no off-flavors were recognized and described in beer “*S. ludwigii* bottled.” Beer “*M. gelida* YGW 184” was evaluated with grades above four for the attribute’s purity of taste, body, carbonation. Beer “*M. gelida* YGW 184” had grades of 3.9 for the attributes aroma (smell) and bitterness. For the attribute aroma (smell) one taster out of eight tasters gave the grade 3 with the off-flavor description diacetyl. Some tasters are very sensitive to diacetyl and can even recognize this sweet-buttery-smelling substance below the usual detection limit in beer (0.1 mg per liter). Overall, seven out of eight tasters did not recognize diacetyl or other off-flavors. This result indicates that consumers would not recognize an off-flavor for this low alcohol beer “*M. gelida* YGW 184.” For the attribute, quality of bitterness of the beer “*M. gelida* YGW 184” one taster out of eight tasters gave the grade 3. The quality of bitterness with a grade 3 means that the bitterness is not harmonic and persists at the back of the mouth and the tongue. Seven out of eight tasters rated the quality of bitterness with the grade 4 which means that the bitterness was harmonious. This result indicates that the bitterness for this low alcohol beer “*M. gelida* YGW 184” would be recognized as harmonious for consumers. The overall sensorial rating of this low alcohol beer “*M. gelida* YGW 184” can be regarded as an average rating for a beer without having any special characteristics and some minor negative characteristics that are not recognized by an “untrained consumer in sensorial analysis.”

**TABLE 7:** Sensory analysis results according to DLG scheme (2.6), in which the beer is judged by its aroma, taste, carbonation, body, and bitterness in a range of 1–5, 1 being the lowest value (negative), 5 being the highest value (positive); arithmetic mean of results of eight tasters.

	*M. gelida* YGW 184 bottled	*S. ludwigii* bottled
Aroma (smell)	3.9	4.3
Purity of taste	4.1	4.5
Body	4.8	4.6
Carbonation	4.9	4.9
Quality of bitterness	3.9	4.0

**FIGURE 3:**
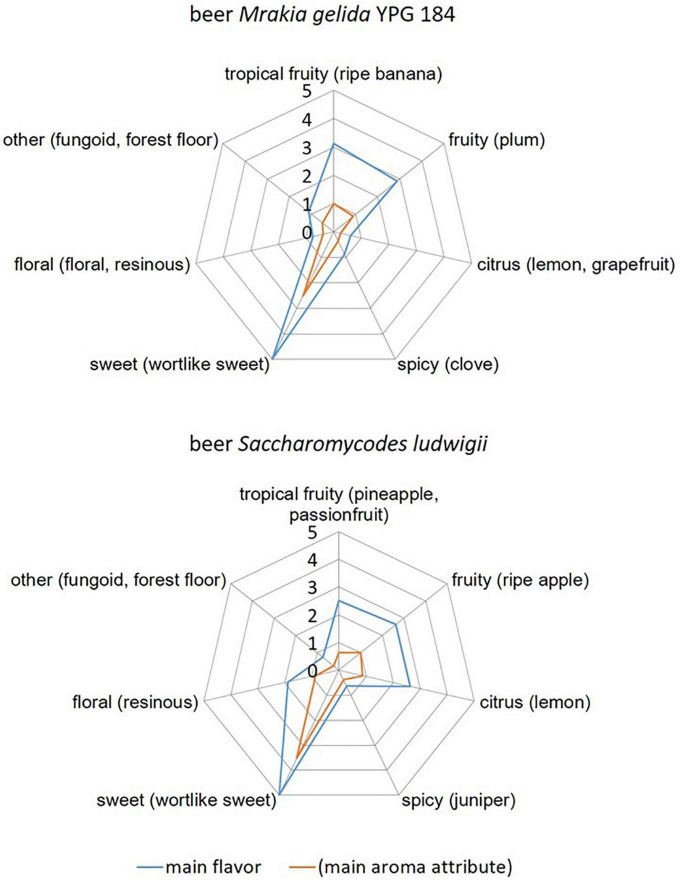
Comparison of the flavors grouped according to the main flavors (sum parameter) and the respective main aroma attribute for beer produced with the yeast strains *Mrakia gelida* YGW 184 **(top)** and *Saccharomycodes ludwigii* **(bottom)**.

Results of a sensory scheme with a focus on yeast-derived aromas according to Meier-Dörnberg et al. (2017) are shown in Figure 3. The two beers produced with *M. gelida* YGW 184 (top) and *S. ludwigii* (bottom) have similar aroma profiles but differ in certain categories. Tasters recognized tropical fruity, sweet, and fruity attributes for both beers. More tasters recognized citrus and floral attributes in beer *S. ludwigii* than in beer *M. gelida* YGW 184. Only single tasters recognized phenolic attributes and other attributes (e.g., fungoid, forest floor). The significance level of the following main aroma attributes was of α = 0.05 according to the method pairwise test of MEBAK sensory 3.1.1 and DIN EN ISO 5495:2007: fruity, sweet in beer *M. gelida* YGW 184 and sweet in beer *S. ludwigii*. The presence of the aroma attributes wort-like and honey was tasted in beer *S. ludwigii* with a significance level of 0.05, whereas the presence of those aroma attributes was not significant for beer *M. gelida*. An absence of the main flavor spicy (consisting of aroma attributes clove, juniper, pepper, and cinnamon) was tasted for beer *M. gelida* YGW 184 with a significance level 0.05. No other main flavors had significant results in the sensory analysis according to the mentioned method. In summary, both beers were very similar with a decent fruitiness and a remaining sweetness. The aroma attributes honey and wort-like within the main flavor category sweet were significantly tasted in the beer produced with *S. ludwigii*, whereas not in the beer produced with *M. gelida*, which indicates that reduction of sweet, wort-like aromas is more pronounced in *M. gelida* wort fermentation than in *S. ludwigii* wort fermentation. *S. ludwigii* had higher rates in citric aroma attributes, which were statistically not significant.

### 3.6. Hygienic characteristics

#### 3.6.1. Biofilm production

Almost all microbes produce biofilm, and the more complex the forming biofilms are, the more difficult it is to eliminate them. The tolerance of microbes to various stressors, such as disinfectants and antibiotics, increases many times over in the form of biofilm, which is why mechanical washing is the most effective way to remove biofilms. Biofilms are viscoelastic and can move and divide, if the situation so requires, for example, due to a lack of nutrients, and proceed elsewhere. Thus, knowledge of microbial biofilm production is important to ensure adequate hygiene management and to know the risks of production.

After 1 day, *M. gelida* was better able to form a biofilm when no agitation was applied during incubation relative to the reference yeast (see Supplementary Table 1). When agitation was applied, the strain did not have time to grow at all or the attachment was so weak that it may have been removed during the rinsing step. Biofilm production after 4 days was intermediate relative to the reference yeasts under both conditions. Thus, the yield of *M. gelida* biofilm does not differ significantly from that of the reference yeasts in these circumstances. Compared to *S. ludwigii*, *M. gelida* poses a lower risk for biofilm production.

#### 3.6.2. Temperature

Temperature plays a major role in the ability of yeasts to grow and maintain their function. Here, the yeasts’ ability to grow at low temperatures was investigated, as well as their ability to grow at 37°C, a property that influences the risk of infections in humans. Temperature tests showed *M. gelida*’s relatively strong performance in cold conditions. Compared to the reference yeasts, its growth was significantly higher at 4°C and 1°C (refrigerator and cold storage conditions). *S. pastorianus* growth at 4°C was weak and *S. ludwigii* did not grow at all. Reference strains showed no growth at 1°C. Only *S. ludwigii* showed slight growth at 37°C (Supplementary Figure 2).

#### 3.6.3. Preservatives

**FIGURE 4:**
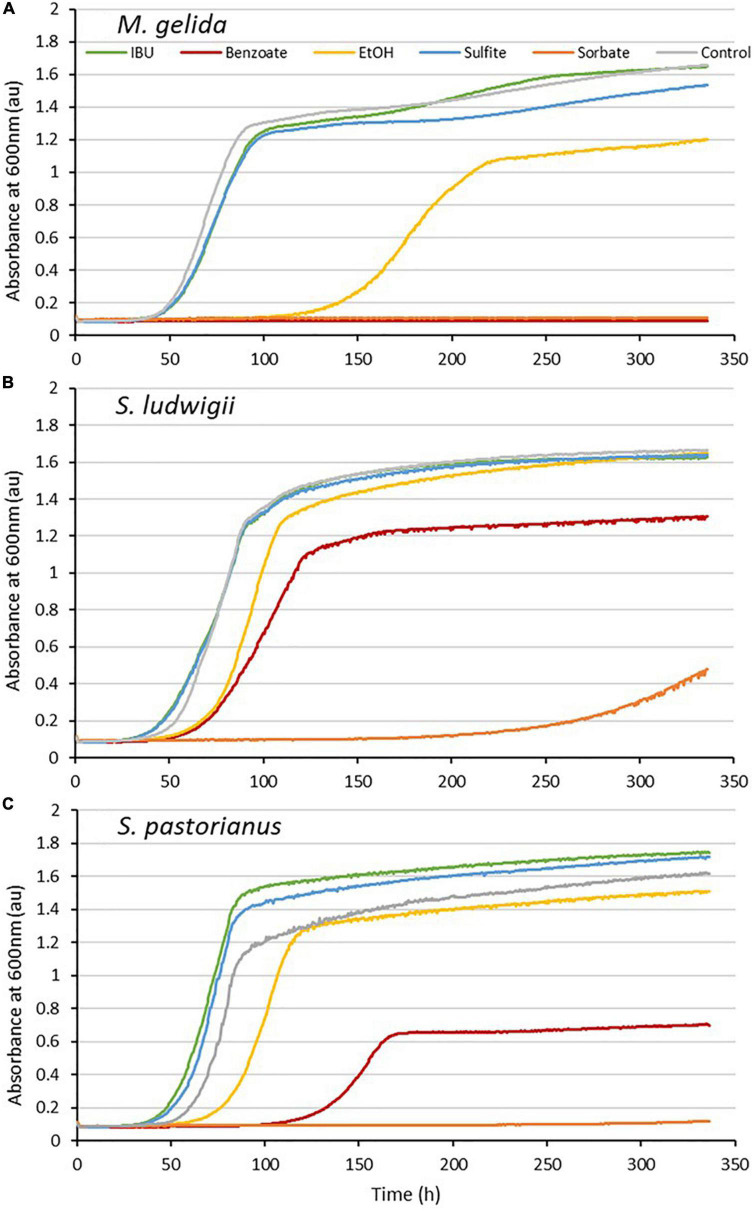
Growth curves with IsoHop (IBU), benzoate, ethanol, sulfite, sorbate and control for *Mrakia gelida* **(A)**, *Saccharomycodes ludwigii* **(B)**, and *S. pastorianus* **(C)**. The x-axis indicates time (h), and the y-axis shows the yeast absorbance at 600 nm (au).

*M. gelida* growth was completely inhibited by the common beverage preservatives sorbate and benzoate. Sulfite delayed growth by 14.5 h and the growth rate decreased by only 14%. Ethanol delayed growth by 48 h and the growth rate decreased by 57%. Thus, *M. gelida*’s growth is moderately sensitive to ethanol (Figure 4). *M. gelida* was clearly more sensitive to the preservatives than the reference yeasts.

#### 3.6.4. Disinfectant tolerance

*S. pastorianus* withstood the disinfectant only weakly in the “unclean condition” at the beginning and was inhibited completely after 5 min. *S. ludwigii* had weak growth at all times in unclean conditions. Both reference yeasts failed to grow in “clean conditions.” *M. gelida* showed no tolerance to the disinfectant in either the unclean or clean conditions, indicating that its use in production does not involve high risks of contamination if proper hygiene management is maintained (Supplementary Figure 2).

#### 3.6.5. Antibiotic resistance

All nine antifungals inhibited *M. gelida*’s growth completely, so there are no clinical risks with *M. gelida*. Reference yeasts were not tested in this research.

## 4. Discussion

*Mrakia* species have been found in arctic, glacial, and alpine habitats such as soils of Antarctica (Di Menna, 1966; Xin and Zhou, 2007), glaciers (Depriest et al., 2000; de García et al., 2007), ice and melting waters (Margesin et al., 2002; Turchetti et al., 2008). In Antarctica, *Mrakia* and closely related genera have been found as dominant micro-organisms in the soils (Margesin and Miteva, 2011). They have remarkable adaptation strategies to survive in the cold environments, such as cold-active enzymes which are also of biotechnological potential (Buzzini et al., 2012). In the context of low-alcohol brewing, this psychrophilly is a particularly advantageous property. Lowering temperature is a routine means to reduce microbial spoilage, and *M. gelida*’s ability to actively ferment at low temperature permits a continuously cool production process–from yeast pitching through to packaging. In contrast, many maltose-negative yeasts that have been used, or considered for use in brewing, are mesophilic and require relatively high fermentation temperatures. *S. ludwigii*, the yeast most commonly used for the production of low-alcohol beers, is for example quite cold-sensitive and grows poorly at 15°C or below (Johansson et al., 2021). In the present study, the cold sensitivity of *S. ludwigii* was particularly apparent in the pilot-scale fermentation. At the relatively warm temperatures used in breweries for fermentation with maltose-negative yeasts, there is a distinct risk of contamination. This risk may be mitigated through the use *M. gelida* or other cold-tolerant yeast at low fermentation temperatures. Use of maltose-negative yeasts for low-alcohol beer brewing is an approach that is feasible for smaller brewing companies without the capacity to invest in dealcoholization equipment (Yabaci Karaoglan et al., 2022). The approach is made even more attractive through the use of a cold-tolerant yeast, as many smaller breweries are capable of maintaining cold temperatures throughout fermentation and downstream processing.

All *M. gelida* strains tested here were capable of fermentation in 7.5°P wort. Results indicate that the yeast were only able to utilize the monosaccharide sugars present, as shown previously be De Francesco et al. (2018). This is supported by low alcohol levels of approx. 0.7% ABV. Reaching a target value of 0.5% ABV can be readily achieved by dilution or by using a weaker wort, e.g., 6.5°P. Likewise, future iterations of the process could involve lowering pH of the wort through the use of lactic acid or similar process with the aim of producing a beer with pH value of <4.5. In the current work, the pH level of the wort was not adjusted to levels typical of finished beers (< pH 4.5) and therefore beers had a quite high pH value of approx. 4.7.

The feasibility of using any new yeast strain for brewing is dependent on the flavor profile of the beers produced. To this end, both chemical and sensorial analyses were performed on the resultant *M. gelida* beers. Levels of yeast-derived higher alcohols and esters were low in both test and reference strains, indicating minimal addition of fruit/floral flavors to beers. Yeast contributes to beer flavor not only by producing volatile aroma compounds, but also by removing the aldehydes that contribute to the worty or grainy flavors, and which are common in beers generated using limited fermentation. In this regard, all yeast strains tested were efficient reducers of wort aldehydes. The test strains appeared to be more efficient at aldehyde reduction than the reference yeast *S. ludwigii*. Results indicate that the *M. gelida* isolates may be particularly suited for the removal of the “worty” flavors. Of note here is that concentrations of methional, which typically imparts a cooked potato aroma, were above the flavor threshold in *S. ludwigii* beers, but below this concentration in the *M. gelida* beers. The particularly “clean” flavor profile of beer produced with *M. gelida* means that this could serve as a base beer for further development. Recent research has indicated that high-quality, low-alcohol beers can be created through blending with full-strength beers, or the judicial use of dry hopping (Rettberg et al., 2022). Such approaches could likewise be used to further develop the taste profile of *M. gelida* beers.

Stress tolerance is an important characteristic of any strain adopted for use in brewing. This is because, firstly, a batch of yeast must be able to survive and function through multiple fermentations and, secondly, because a yeast should be controllable and not pose a risk to brewery operations or to the health of those handling the yeast. Regarding the former case, there was no indication that the fermentation conditions employed in these trials were stressful for the yeast. All test strains were capable of growth and maintained a high viability (>90%)–making them suitable for re-use in subsequent fermentations. Regarding control *M. gelida*, the yeast was found to be sensitive to the disinfectant Oxonia (peroxyacetic acid). Sensitivity was greater than that of the reference yeasts *S. pastorianus* and *S. ludwigii*. *M. gelida* was likewise more sensitive to a range of preservative compounds relative to the reference yeasts. Sorbate and benzoate prevented growth completely. Growth was possible in the presence of sulfite and ethanol, but in both cases the onset of growth was delayed significantly, and growth rate was reduced. There is therefore no indication that preservative tolerance of *M. gelida* would pose any risk to brewery operations. Additionally, the biofilm-forming potential of *M. gelida* was found to be limited, and lower than that of *S. ludwigii*. It can be assumed therefore that no additional tolerance could be achieved through this means. The strain’s tolerance of hop bitter acids is a potential advantage if increased hop dosage, or dry-hopping, is considered as a way to enhance the flavor profile of the beer. With respect to the risk to human health, there is no known case of a *Mrakia* species causing a disease in humans. Infection by *M. gelida* is in any case precluded by the species’ inability to grow at human body temperature. Furthermore, *M. gelida* strain showed no ability to grow when challenged with common anti-mycotic agents, meaning that an infection would be readily treatable. In the laboratory safety studies with the beers produced with different *Mrakia* species (*Mrakia robertii* sp. nov., *Mrakia blollopis* sp. nov., and *Mrakiella niccombsii* sp. nov.), no abnormalities in the laboratory rats were observed (Thomas-Hall et al., 2010). It can be said therefore that with this yeast the risk to the brewery operations or operators is minimal. On the contrary, the sensitivity of the yeast to even moderately high temperatures may be a disadvantage as there is a risk to viability when a cold-chain in the brewery cannot be maintained.

*Mrakia gelida* is not on the GRAS (i.e., Generally Recognized As Safe) list of organisms updated by the United States Food and Drug Administration (FDA), nor on the qualified presumption of safety (QPS) list of the European Food Safety Authority (EFSA) (last updated 7/2022). Earlier, there has not been enough information about the characteristics of the species nor information to offer because breweries have not been familiar with the potential of *M. gelida*. If the consumer-safety/acceptability of products fermented with *M. gelida* were to be assessed, the following aspects will be considered: According to EFSA, the species must not be resistant to antifungal substances. Another aspect of acceptability is that the yeast can be used in the production processes, if there are no longer any viable cells in the final product. On the other hand, the number of living cells in the final products made with *M. gelida* should not be a problem as it cannot grow at the temperatures of the human digestive tract.

Results of this study support the contention of De Francesco et al. (2018) and Yabaci Karaoglan et al. (2022) that *M. gelida* is a promising species with respect to low-alcohol brewing. We further show that this potential does not appear to be strain-specific, as all *M. gelida* strains tested here had similar functional properties. Additional tests showed that any risk contamination or infection was negligible. Results suggest that the species is suitable for the production of clean tasting low-alcohol beers at temperatures that would prohibit the growth of contaminant yeasts or bacteria. Fermentation with *M. gelida* therefore represents a reliable, safe, and relatively low-cost method for producing low-alcohol beers, and may be particularly attractive for those brewers that do not have access to dealcoholization equipment.

## Data availability statement

The datasets presented in this study can be found in online repositories. The names of the repository/repositories and accession number(s) can be found in the article/Supplementary material.

## Author contributions

RL and PV: conceptualization, strain isolations, and original draft preparation. RL and MC-E: strain identifications. FM: fermentation trials and analysis of fermentation data. BG: conceptualization, original draft preparation, supervision, and methodology. RE: hygienic assays, analysis of data, and original draft preparation. MH: sensory and aroma analysis on the bottled beers, original draft preparation, and conceptualization. EJ and LH: original draft preparation. TJ: funding acquisition, project administration, and editing and finalization of the manuscript. All authors contributed to the article and approved the submitted version.

## Footnotes

1
https://blast.ncbi.nlm.nih.gov/Blast.cgi
