# Supplementary material for: Brewing potential of strains of the boreal wild yeast Mrakia gelida
Source: Front Microbiol. 2023 Feb 9;14:1108961. doi: 10.3389/fmicb.2023.1108961 (PMC9947644; doi:10.3389/fmicb.2023.1108961)

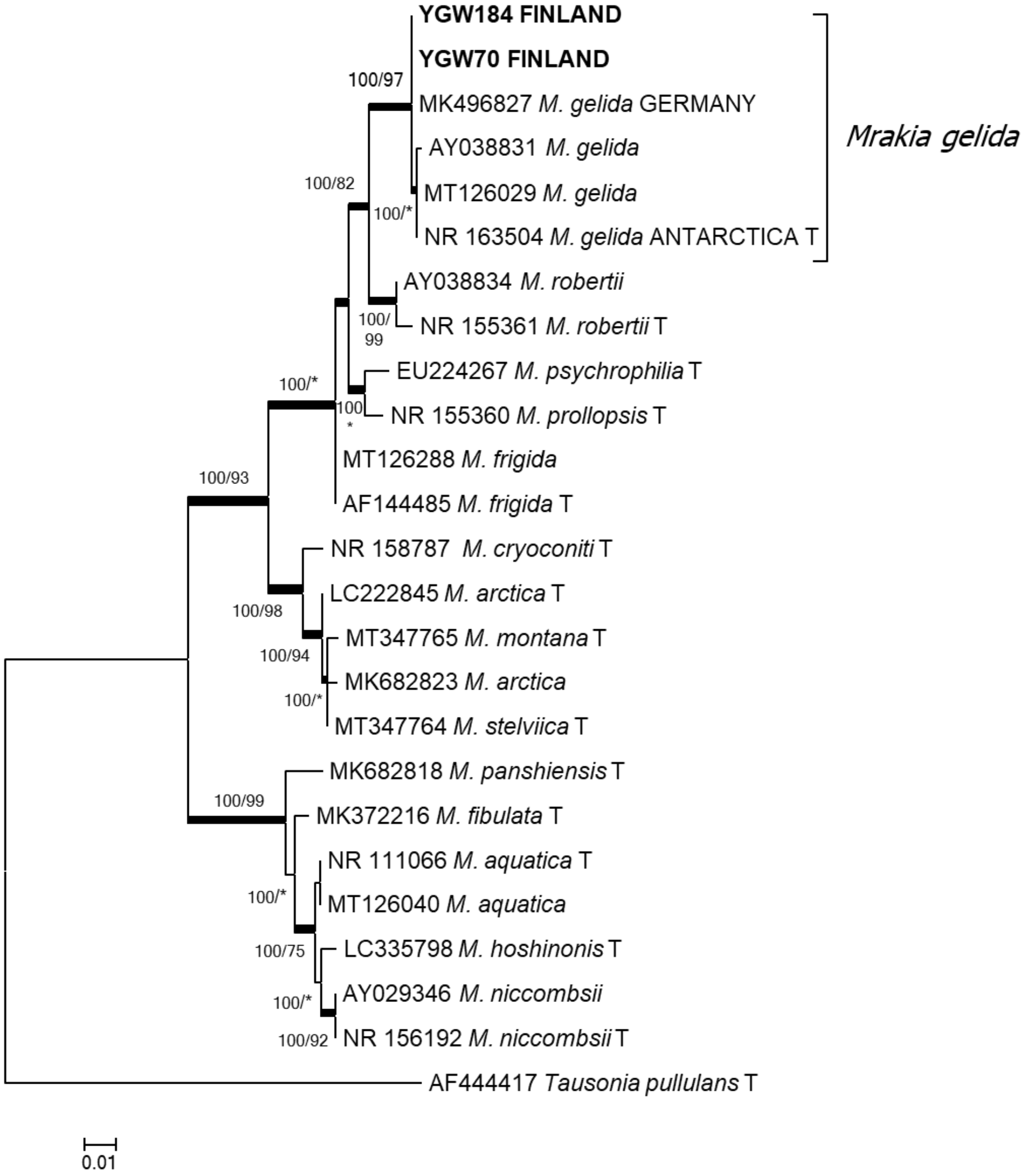

Supplementary Figure S1. Phylogenetic tree of *Mrakia* obtained from PhyML (ML) analyses of the ITS data set. Isolates used for DNA sequencing in this study are printed in bold type. Bootstrap support values above 70% for ML /Maximum Parsimony (MP) are presented at the nodes. Posterior probabilities (above 70%) obtained from Bayesian (BI) are indicated by bold lines at the relevant branching points. *Bootstrap values <70 %. T ex-type isolate of *Mrakia gelida*. Scale bar = total nucleotide difference between taxa.

Supplementary Table S1. Average biofilm formation capacity of *M. gelida*, *S. ludwigii*, and *S. pastorianus* in 24 h and 4 d tests with and without agitation.

Biofilm forming	No agitation		Agitation	
	24 h	4 days	24 h	4 days
Mrakia gelida	0.014	0.44	-0.0010	0.58
S. ludwigii	0.0065	0.80	0.027	0.83
S. pastorianus	0.0050	0.28	0.010	0.22

Supplementary Figure S2. Disinfect and temperature test results: (A) *M. gelida* growth with P3-oxonia active (left clean; 0.3 % BSA and right unclean; 3 % BSA); (B) Growths at 4°C for 2 weeks (left pictures, top *M. gelida*, middle *S. ludwigii*, and bottom *S. pastorianus*) and 1°C for three weeks (right pictures, top *M. gelida*, middle *S. ludwigii*, and bottom *S. pastorianus*); (C) Growths at 37°C for 3 days: *M. gelida* top, *S. ludwigii* middle, and *S. pastorianus* bottom. *M. gelida* did not grow at all at 37°C, which was expected for psychrotolerant yeasts. *S. ludwigii* (middle picture) grow slightly at 37°C, and *S. pastorianus* did not grow at all in this temperature.

A

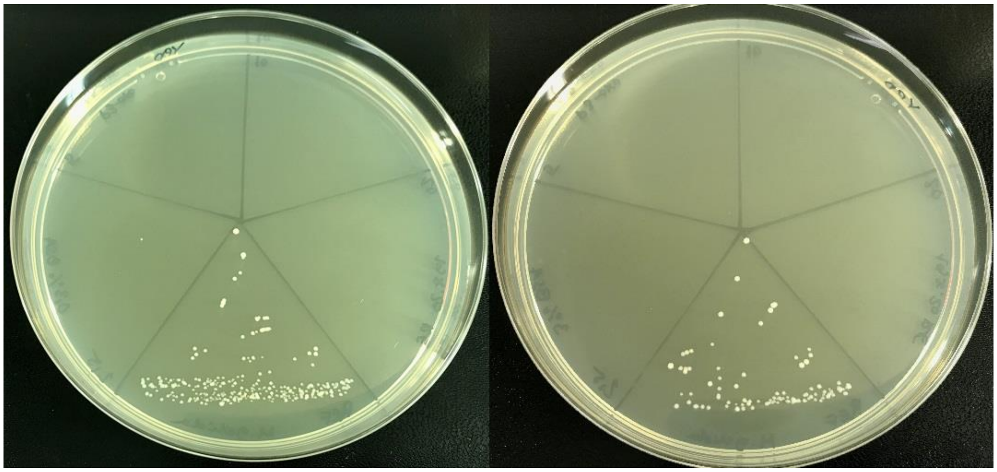

B

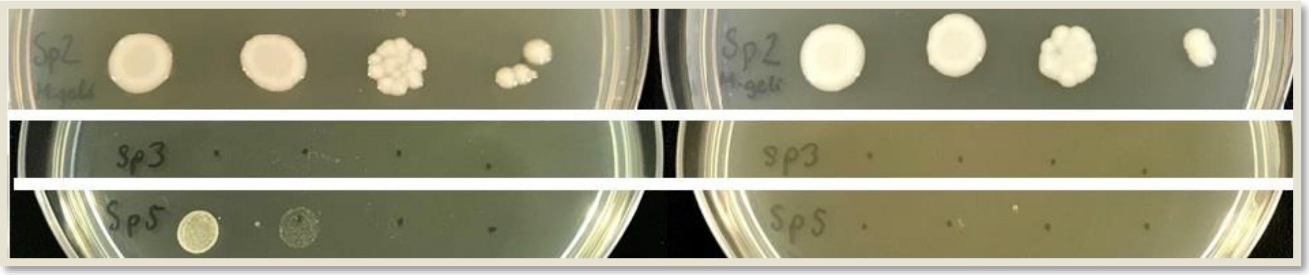

C

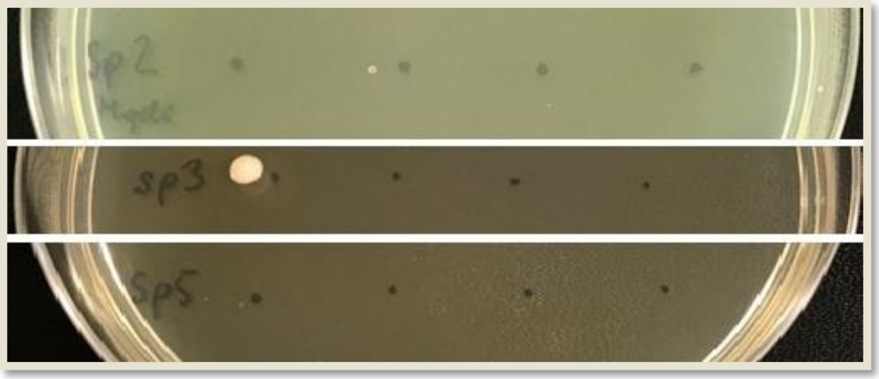

Supplement: Supplementary file 1 [file Data_Sheet_1.PDF]
